# Impact of Obesity on Joint Replacement Surgery Outcomes: A Comparative Study

**DOI:** 10.7759/cureus.80623

**Published:** 2025-03-15

**Authors:** Abdul Rahman, Hafiz Muhammad Abid Hasan, Rahman Ali, Hidayat Ullah, Saeed Ahmad, Muhammad Saqib

**Affiliations:** 1 Orthopaedic Surgery Unit One, Jinnah Hospita Lahore, Allama Iqbal Medical College, Lahore, PAK; 2 Department of Orthopaedic Surgery, Tehsil Head Quarter (THQ) Zafarwal District, Narowal, PAK; 3 Department of Orthopaedics, Salman Medical and Teaching Hospital, Swat, PAK; 4 Department of Orthopaedics, Peshawar Medical College and Affiliated Hospitals, Peshawar, PAK; 5 Department of Trauma and Orthopaedics, Ghurki Trust Teaching Hospital, Lahore, PAK; 6 Department of Orthopaedics, Gajju Khan Medical College, Swabi, PAK

**Keywords:** complications, joint replacement surgery, mobility, obesity, osteoarthritis, postoperative outcomes

## Abstract

Background: Obesity negatively impacts joint health and poses challenges during joint replacement surgery, leading to less favorable postoperative outcomes.

Objective: This study aims to assess the impact of obesity on postoperative outcomes following joint replacement surgery by comparing obese and non-obese patients.

Methodology: This prospective observational study was conducted from January 2022 to December 2023, including 186 patients, of which 93 were classified as obese (BMI > 30 kg/m²) and 93 as non-obese (BMI < 30 kg/m²). Data on postoperative complications, mobility, pain scores, comorbidities, and demographics were collected and analyzed using IBM SPSS Statistics for Windows, Version 25.0 (Released 2017; IBM Corp., Armonk, New York, United States). Multivariate logistic regression was employed to identify independent predictors of adverse outcomes.

Results: The mean BMI was significantly higher in obese patients (34.8 ± 3.2 kg/m²) compared to non-obese patients (24.6 ± 2.1 kg/m²). Obese patients had a higher prevalence of severe osteoarthritis (45.16% vs. 26.88%, p = 0.008), longer hospital stays (7.6 ± 2.1 vs. 5.4 ± 1.7 days, p < 0.001), and an increased incidence of postoperative complications, including surgical site infections (11.83% vs. 4.30%, p = 0.05). Multivariate analysis revealed that obesity was an independent predictor of poor postoperative outcomes (OR: 2.40, 95% CI: 1.30-4.50, p = 0.005).

Conclusion: Obese patients experienced higher complication rates, greater postoperative discomfort, reduced mobility, and prolonged hospital stays compared to non-obese patients. These findings highlight the significant negative impact of obesity on joint replacement surgery outcomes and emphasize the need for tailored preoperative assessments, optimized perioperative care, and targeted rehabilitation strategies for obese patients.

## Introduction

The incidence of obesity is increasing alarmingly in both developed and developing countries, making it a worldwide health issue [1]. Obesity is defined as an excessive build-up of body fat and is strongly associated with several comorbidities such as diabetes, respiratory disorders, and cardiovascular disease [2]. However, obesity's negative effects on the musculoskeletal system, especially joint health, are among its most serious but often overlooked effects [3]. The mechanical stress on weight-bearing joints, such as the knees and hips, is greatly increased by excess body weight, which speeds up articular cartilage degradation and aids in the development and progression of osteoarthritis (OA) [4].

In addition to mechanical strain, obesity exacerbates joint injury by causing systemic inflammation via the production of pro-inflammatory cytokines and adipokines [5]. This combined load of biomechanical and biochemical stress not only makes joint degradation more likely but also makes pain worse and limits mobility, which lowers the overall quality of life of those who are affected [6]. The demand for joint replacement procedures, especially complete knee and hip arthroplasties, has increased in tandem with the rising obesity rates [7].

Obesity poses particular difficulties for joint replacement results, even with improvements in surgical methods and prosthetic design [8]. Infection, poor wound healing, prosthesis loosening, and decreased postoperative mobility are among the complications that obese patients often experience at greater rates [9]. Additionally, obesity may impede postoperative rehabilitation and delay functional recovery [10]. These problems not only make it more difficult for patients to get better after surgery but also make hospital stays longer and the number of revisions higher [11].

Understanding how excess body weight impacts the course of joint illness and the results of joint replacement surgery is crucial given the intricate relationship between obesity and joint health. In order to help doctors, enhance preoperative evaluations, maximize surgical results, and customize postoperative care for obese patients, this study aims to investigate these dynamics. The objective of the study was to evaluate the impact of obesity on joint health and assess postoperative outcomes following joint replacement surgery in obese patients compared to non-obese individuals.

## Materials and methods

This research was carried out in accordance with the Strengthening the Reporting of Observational Studies in Epidemiology (STROBE) standards to guarantee meticulous and honest reporting of observational studies. This prospective observational study, conducted at Gajju Khan Medical College (GKMC), Swabi, Pakistan, from January 2022 to December 2023, aimed to assess the impact of obesity on joint replacement surgery outcomes. Specifically, the study compared perioperative complications, postoperative recovery, hospital stay duration, and surgical site infections between obese and non-obese patients undergoing joint replacement surgery. The study received ethical approval from the Institutional Review Board of GKMC (approval number: DIR/GKMC/DO/228).

Inclusion and exclusion criteria

Inclusion Criteria

Patients aged ≥18 years with a recorded body mass index (BMI) undergoing primary total knee or hip replacement surgery were included. Participants were categorized based on the World Health Organization (WHO) classification as non-obese (BMI < 30 kg/m²) and obese (BMI ≥ 30 kg/m²) [12]. 

Exclusion Criteria

The study excluded patients with a history of prior joint replacement, inflammatory joint conditions (e.g., rheumatoid arthritis), cancer, or systemic disorders that could impact surgical outcomes. However, other systemic disorders such as chronic kidney disease, liver diseases, neurological disorders, and autoimmune conditions (excluding rheumatoid arthritis) were not explicitly mentioned in the exclusion criteria. These conditions were not included due to their varied impact on surgical outcomes, which were beyond the primary scope of the study focusing on obesity-related joint health and postoperative recovery.

Sample size

A total of 186 patients were selected using convenience sampling. All eligible patients who underwent joint replacement surgery at GKMC during the study period and provided informed consent were included.

Data collection

Direct interviews, postoperative follow-ups, and patient medical records were used to collect data. Preoperative assessments included demographics, BMI, comorbidities, and joint-specific clinical evaluations. Functional mobility before surgery was measured using the Timed-Up-and-Go (TUG) test (recorded in seconds, with lower scores indicating better mobility) and the De Morton Mobility Index (DEMMI) (scored in points, with higher scores reflecting better mobility), as described by Van der Sluis et al. [13]. Evaluations were conducted at one, six, and 12 months post surgery, documenting postoperative outcomes such as complication rates, hospital stay duration, pain levels, mobility test results, and patient-reported functional outcomes over a 12-month follow-up period.

During patient examinations and interviews, researchers explained the purpose and content of the questionnaire before administering it. The Western Ontario and McMaster Universities Osteoarthritis Index (WOMAC) questionnaire [14] was then completed by researchers based on patient responses. This validated instrument consists of 24 items categorized into three subscales: pain (five items), stiffness (two items), and physical function (17 items). Patients were assessed at baseline (pre-surgery) and at follow-up visits to track symptom progression and functional recovery. The total WOMAC score was calculated, with higher scores indicating greater impairment.

Definitions

(i) Surgical site infection (SSI) was defined as infection occurring at the surgical incision site within 30 days postoperatively, classified as superficial (skin and subcutaneous tissue) or deep (involving prosthesis or joint space) [15]. (ii) Wound healing delay was defined as prolonged wound closure requiring additional medical interventions such as debridement or extended dressing changes [16]. (iii) Prosthetic loosening was defined as radiographic or clinical evidence of implant instability, leading to pain or functional limitations [17].

Statistical analysis

The IBM SPSS Statistics for Windows, Version 25.0 (Released 2017; IBM Corp., Armonk, New York, United States) was used to analyze the data. For demographic factors, descriptive statistics were used. The chi-square test was used to compare categorical data, and independent t-tests were used to evaluate continuous variables when needed. To find variables that are independently linked to postoperative outcomes, a multivariate logistic regression analysis was performed. P-values below 0.05 were regarded as statistically significant.

## Results

The average age of obese patients was 62.3 ± 8.5 years, whereas that of non-obese patients was 60.7 ± 7.9 years (Table 1). The distribution of genders was comparable across the groups: the obese group included 45 (48.39%) male and 48 (51.61%) female participants, whereas the non-obese group had 47 (50.54%) male and 46 (49.46%) female participants. Obese patients had a substantially higher mean BMI (34.8 ± 3.2 kg/m²) than non-obese people (24.6 ± 2.1 kg/m²). Compared to those who were not obese, obese patients had higher incidences of cardiovascular disease (n=26, 27.96% vs. n=19, 20.43%), diabetes mellitus (n=41, 44.09% vs. n=28, 30.11%), and hypertension (n=58, 62.37% vs. n=37, 39.78%).

**Table 1:** Baseline Characteristics of Study Participants (N=186)

Variable		Obese Patients (n = 93)	Non-Obese Patients (n = 93)
Age	Mean ± SD	62.3 ± 8.5 years	60.7 ± 7.9 years
Gender	Male, n (%)	45 (48.39%)	47 (50.54%)
	Female, n (%)	48 (51.61%)	46 (49.46%)
BMI	Mean ± SD	34.8 ± 3.2 kg/m²	24.6 ± 2.1 kg/m²
Comorbidities	Hypertension, n (%)	58 (62.37%)	37 (39.78%)
	Diabetes Mellitus, n (%)	41 (44.09%)	28 (30.11%)
	Cardiovascular Disease, n (%)	26 (27.96%)	19 (20.43%)

Obese individuals showed a statistically significant greater prevalence of severe osteoarthritis (n=42, 45.16%) than non-obese patients (n=25, 26.88%) (p = 0.008), as shown in Table 2. The prevalence of moderate osteoarthritis was comparable between obese and non-obese groups (n=39, 41.94% vs. n=44, 47.31%) (p = 0.45), indicating no statistically significant difference. Conversely, mild osteoarthritis was significantly more frequent in non-obese individuals (n=24, 25.81%) compared to obese patients (n=12, 12.90%) (p = 0.03), suggesting a higher proportion of early-stage disease in the non-obese cohort. The mean preoperative pain levels (visual analog scale (VAS)) were greater in obese individuals (7.8 ± 1.1) than in non-obese patients (6.5 ± 1.3) (p < 0.001). Furthermore, obese patients had a substantially lower mean preoperative mobility score (3.2 ± 0.9) than non-obese people (4.1 ± 1.0; p < 0.001).

**Table 2:** Preoperative Joint Health Assessment (N=186) VAS: visual analog scale

Assessment Parameter		Obese Patients (n = 93)	Non-Obese Patients (n = 93)	p-value
Osteoarthritis Grade	Mild, n (%)	12 (12.90%)	24 (25.81%)	0.03
	Moderate, n (%)	39 (41.94%)	44 (47.31%)	0.45
	Severe, n (%)	42 (45.16%)	25 (26.88%)	0.008
Preoperative Pain Score (VAS)	Mean ± SD	7.8 ± 1.1	6.5 ± 1.3	<0.001
Preoperative Mobility Score	Mean ± SD	3.2 ± 0.9	4.1 ± 1.0	<0.001

According to the postoperative results, obese patients reported significantly higher pain levels at all follow-up intervals compared to non-obese patients. The mean VAS scores for obese patients were 6.2 ± 1.0 at one month, 4.9 ± 1.1 at six months, and 4.1 ± 1.0 at 12 months, whereas non-obese patients had mean scores of 4.8 ± 0.9, 3.5 ± 0.8, and 3.2 ± 0.8, respectively (all p < 0.001), as shown in Table 3. Similarly, obese patients demonstrated significantly lower mobility scores at all time points (all p < 0.001): 3.5 ± 1.0 at one month, 4.5 ± 1.1 at six months, and 5.0 ± 1.1 at 12 months, compared to 4.5 ± 0.9, 5.5 ± 1.0, and 6.1 ± 0.9, respectively, in non-obese patients.

**Table 3:** Postoperative Outcomes Over Time (N=186) t: t-tests; χ²: Chi-square tests; CI: confidence interval, VAS: visual analog scale

Outcome Measure		Obese Patients (n = 93)	Non-Obese Patients (n = 93)	p-value	Test Statistic	95% CI (Lower bound, Upper bound)
Pain Score (VAS), mean ± SD	1 Month	6.2 ± 1.0	4.8 ± 0.9	<0.001	t = 8.43	1.03, 1.78
	6 Months	4.9 ± 1.1	3.5 ± 0.8	<0.001	t = 9.12	1.06, 1.82
	12 Months	4.1 ± 1.0	3.2 ± 0.8	<0.001	t = 6.77	0.63, 1.16
Mobility Score, mean ± SD	1 Month	3.5 ± 1.0	4.5 ± 0.9	<0.001	t = -7.63	-1.24, -0.74
	6 Months	4.5 ± 1.1	5.5 ± 1.0	<0.001	t = -7.02	-1.33, -0.78
	12 Months	5.0 ± 1.1	6.1 ± 0.9	<0.001	t = -7.46	-1.46, -0.81
Surgical Site Infection (SSI), n (%)	Superficial SSI	6 (6.45%)	2 (2.15%)	0.12	χ² = 2.41	0.91, 5.26
	Deep SSI	5 (5.38%)	2 (2.15%)	0.18	χ² = 1.77	0.79, 5.84
Delayed Wound Healing, n (%)		9 (9.68%)	3 (3.23%)	0.04	χ² = 4.22	[1.05, 8.38]
Prosthetic Loosening, n (%)		6 (6.45%)	2 (2.15%)	0.15	χ² = 2.06	[0.86, 6.57]
Length of Hospital Stay (days), mean ± SD		7.6 ± 2.1	5.4 ± 1.7	<0.001	t = 7.49	[1.60, 2.88]
Revision Surgery Required (%), n (%)		5 (5.38%)	1 (1.08%)	0.04	χ² = 4.19	[1.01, 7.86]
Reasons for Revision Surgery, n (%)	Infection-Related	3 (3.23%)	1 (1.08%)	0.07	χ² = 3.29	[0.94, 8.12]
	Aseptic Loosening	2 (2.15%)	0 (0%)	0.1	χ² = 2.69	[0.78, 7.34]

Postoperative complications were more prevalent in obese patients, including surgical site infections (11.83% vs. 4.30%, p = 0.05), delayed wound healing (9.68% vs. 3.23%, p = 0.04), and prosthetic loosening (6.45% vs. 2.15%, p = 0.15). Additionally, the need for revision surgery was significantly higher among obese patients (5.38% vs. 1.08%, p = 0.04), with infection-related causes being the most frequent reason for revision. Moreover, obese patients had a significantly longer hospital stay (7.6 ± 2.1 days vs. 5.4 ± 1.7 days, p < 0.001). These findings highlight the increased postoperative risks and poorer functional recovery associated with obesity in patients undergoing joint replacement surgery.

According to Table 4, obese individuals had a lower preoperative mobility score (3.2 ± 0.9) than non-obese patients (4.1 ± 1.0) (p < 0.001), according to the comparison of functional outcomes. Both groups showed improvement after a year, although the mobility scores of obese patients remained lower (5.0 ± 1.1) than those of non-obese people (6.1 ± 0.9) (p < 0.001). Non-obesity patients showed a substantially larger increase in quality of life (83.87%) than obese patients (66.67%) (p = 0.006), despite obese patients experiencing greater pain reduction (VAS improvement of 3.7 ± 1.0) than non-obese patients (3.3 ± 0.8) (p = 0.02).

**Table 4:** Comparison of Functional Outcomes (Preoperative vs. Postoperative)

Outcome	Obese Patients (n = 93)	Non-Obese Patients (n = 93)	p-value
Preoperative Mobility Score, mean ± SD	3.2 ± 0.9	4.1 ± 1.0	<0.001
12-Month Mobility Score, mean ± SD	5.0 ± 1.1	6.1 ± 0.9	<0.001
Improvement in Pain Score (VAS), mean ± SD	3.7 ± 1.0	3.3 ± 0.8	0.02
Improvement in Quality of Life (%), n (%)	62 (66.67%)	78 (83.87%)	0.006

The multivariate analysis revealed that obesity (BMI ≥ 30 kg/m²) was a significant independent predictor of worse postoperative outcomes, with an odds ratio (OR) of 2.40 (95%CI: 1.30-4.50, p = 0.005), as shown in Table 5. Diabetes mellitus was also significantly associated with adverse outcomes (OR: 1.80, 95%CI: 1.00-3.20, p = 0.04). Conversely, better postoperative outcomes were linked to higher preoperative mobility scores, with an OR of 0.70 per unit increase (95%CI: 0.50-0.90, p = 0.01). In contrast, age, gender, and hypertension were not found to be significant predictors of postoperative results, with p-values of 0.24, 0.98, and 0.17, respectively.

**Table 5:** Multivariate Analysis of Factors Affecting Postoperative Outcomes

Variable	Odds Ratio	95% Confidence Interval	p-value
Obesity (BMI ≥ 30 kg/m²)	2.40	1.30 – 4.50	0.005
Age	1.10	0.90 – 1.30	0.24
Gender	1.00	0.60 – 1.70	0.98
Hypertension	1.50	0.80 – 2.70	0.17
Diabetes Mellitus	1.80	1.00 – 3.20	0.04
Preoperative Mobility Score	0.70	0.50 – 0.90	0.01

## Discussion

This study demonstrates the substantial impact of obesity on the results of joint replacement surgery, showing that obese patients had worse postoperative outcomes than non-obese patients. In accordance with previous studies, obese patients demonstrated significantly reduced preoperative mobility scores (3.2 ± 0.9) compared to their non-obese counterparts (4.1 ± 1.0) (p < 0.001), indicating a higher degree of functional limitation prior to surgery [18]. Liao et al. observed similar findings, demonstrating that surgical recovery was delayed and baseline mobility was lower in obese participants [19].

According to our findings, preoperative pain ratings for obese patients were substantially greater (VAS: 7.8 ± 1.1) than those for non-obese people (6.5 ± 1.3) (p < 0.001). This finding aligns with a previous study, which has also identified an association between higher BMI and increased severity of joint pain [20]. Over the course of the follow-up period after surgery, obese patients continued to report greater levels of pain; their 12-month VAS score was 4.1 ± 1.0, whereas that of non-obese patients was 3.2 ± 0.8 (p < 0.001). This enduring discrepancy highlights the long-term effects of obesity on post-operative joint discomfort.

Additionally, the obese group had a greater incidence of complications. Similar to earlier research that found higher infection rates in obese patients undergoing arthroplasty [21], surgical site infections were found in 11.83% of obese patients compared to 4.30% in non-obese persons (p = 0.05). Furthermore, obese patients had higher prosthetic loosening (6.45% vs. 2.15%, p = 0.15) and delayed wound healing (9.68% vs. 3.23%, p = 0.04), which is in line with other studies showing obesity as a risk factor for postoperative complications and prosthesis-related problems [22].

Obesity also significantly hampered functional recovery. At 12 months postoperatively, obese patients had a mobility score of 5.0 ± 1.1, while non-obese patients achieved a score of 6.1 ± 0.9 (p < 0.001), indicating better functional recovery in the non-obese group. Higher BMI was linked to lower functional improvements after total knee arthroplasty, according to prior research that observed similar patterns [23]. These results are corroborated by our multivariate analysis, which demonstrates that obesity was an independent predictor of worse postoperative outcomes (OR: 2.40, 95%CI: 1.30-4.50, p = 0.005). Furthermore, a lower preoperative mobility score (OR: 0.70, p = 0.01) and diabetes mellitus (OR: 1.80, p = 0.04) were significant predictors of unfavorable outcomes, which is consistent with other research that shows these characteristics to be important risk factors for postoperative recovery [24].

Strengths and limitations

One of the key strengths of this study is its prospective observational design, which allowed for comprehensive data collection and a 12-month longitudinal follow-up, providing valuable insights into the long-term effects of obesity on joint replacement outcomes. The inclusion of a well-defined sample with an equal number of obese and non-obese patients enhanced the reliability of comparative analyses. Additionally, the use of standardized outcome measures, such as the VAS for pain and mobility assessment, reinforced the objectivity of the results.

However, this study also has certain limitations. The single-center design may restrict the generalizability of the findings, and the use of convenience sampling could introduce selection bias. Furthermore, potential confounding factors, such as variations in surgical techniques, differences in postoperative rehabilitation protocols, and patient adherence to rehabilitation, may have influenced the results. To minimize these limitations, strict inclusion criteria were followed, standardized surgical and rehabilitation protocols were encouraged, and statistical adjustments were applied to control for potential confounders. Despite these efforts, residual confounding cannot be entirely ruled out.

## Conclusions

Obese patients demonstrated significantly higher rates of postoperative complications, increased pain levels, reduced mobility, and prolonged hospital stays compared to their non-obese counterparts, highlighting the profound negative impact of obesity on joint replacement outcomes. Our findings confirm that obesity, along with diabetes mellitus and poorer preoperative mobility, serves as an independent predictor of adverse postoperative outcomes. The obese patients faced a greater risk of surgical site infections, delayed wound healing, prosthetic loosening, and the need for revision surgery. These results emphasize the necessity of personalized preoperative evaluations, optimized perioperative care, and targeted rehabilitation programs to mitigate risks and enhance postoperative recovery. Implementing tailored strategies for obese patients undergoing joint replacement surgery may lead to improved surgical outcomes and better long-term functional recovery.
